# Acute effects of FLT3L treatment on T cells in intact mice

**DOI:** 10.1038/s41598-022-24126-4

**Published:** 2022-11-14

**Authors:** Gideon Wolf, Allison N. Gerber, Zachary G. Fasana, Kenneth Rosenberg, Nevil J. Singh

**Affiliations:** Department of Microbiology and Immunology, University of Maryland School of Medicine, 685 W Baltimore St., HSF1, Room 380, Baltimore, MD 21201 USA

**Keywords:** Lymphocyte activation, Haematopoietic cell growth factors, T cells

## Abstract

Peripheral T cells express a diverse repertoire of antigen-specific receptors, which together protect against the full range of pathogens. In this context, the total repertoire of memory T cells which are maintained by trophic signals, long after pathogen clearance, is critical. Since these trophic factors include cytokines and self-peptide-MHC, both of which are available from endogenous antigen-presenting cells (APC), we hypothesized that enhancing APC numbers in vivo can be a viable strategy to amplify the population of memory T cells. We evaluated this by acutely treating intact mice with FMS-like tyrosine kinase 3 ligand (Flt3l), which promotes expansion of APCs. Here we report that this treatment allowed for, an expansion of effector-memory CD4+ and CD8+ T cells as well as an increase in their expression of KLRG1 and CD25. In the lymph nodes and spleen, the expansion was limited to a specific CD8 (CD44-low but CD62L−) subset. Functionally, this subset is distinct from naïve T cells and could produce significant amounts of effector cytokines upon restimulation. Taken together, these data suggest that the administration of Flt3L can impact both APC turnover as well as a corresponding flux of specific subsets of CD8+ T cells in an intact peripheral immune compartment.

## Introduction

The ability of the peripheral immune system to consistently mount successful responses hinges on the availability of a sufficiently diverse collection of T cells at all times. Naïve T cells arrive in the secondary lymphoid organs after completing thymic development. When a naïve T cell is activated with cognate antigen, it undergoes transition into an effector phase, characterized by extensive proliferation and cytokine production. This transient change in the number of cells is followed then by a contraction of the responding population, with some memory T cells remaining. At the end of an immune response, cells of the immune system re-establish a homeostatic balance, with a relatively stable proportion of different T cell subsets^1^. The composition of this homeostatically constrained population is important. Naïve T cells are required for generating new responses but maintaining a population of diverse memory T cells allows a rapid adaptive immune response upon subsequent re-exposure to antigen^2,3^. Impairment or depletion of memory T cells can reduce the response to or clearance of a foreign pathogen^4,5^. The memory T cell pool itself can be broken into sub-compartments based on not only expression of extracellular proteins such as CD44, CD62L, and CCR7, but tissue residence and migratory properties ; these include, but are not limited to Central Memory (T_CM_), Effector Memory (T_EM_), and Tissue Resident Memory (T_RM_) cells^6,7^.

There are several physiological phenomena that impact the composition of this peripheral T cell population. Although acute and chronic antigen exposures routinely affects T cell numbers^8^ natural homeostatic processes such as aging impacts both the functionality and diversity of the memory T cell repertoire. Indeed chronic antigen exposure also contributes to the overarching immunological decline termed “immunosenescence”^9–12^. Production of new naïve T cells to the peripheral T cell pool diminishes with age due to factors such as thymic involution, making protective immunity in older individuals more heavily dependent on maintaining a diverse repertoire of memory T cells^7,13^. Viral infections can also cause significant perturbations of the total memory T cell compartment due to cell death and loss in T cell receptor (TCR) diversity^3,14^. In AIDS, this is a major cause of immunosuppression, and subsequent progression of disease severity^15,16^. Importantly, an intuitive treatment strategy for many of these contexts, would be to globally enhance the survival and maintenance of specific sub-populations of T cells—for instance, the memory T cell population. Efforts to develop approaches to accomplish this, however, is limited by our incomplete understanding of naïve and memory T cell homeostasis.

The maintenance of T cells in vivo is thought to require multiple pro-survival or trophic signals. These include signals from the cytokines including IL-7/IL-15 and tonic signals from TCR engagement of self-peptide MHC complexes (spMHC)^17,18^. Conceptually, we can segregate these factors into two broad classes, the first being “public” signals, such as cytokine/nutrient-mediated signaling, since all T cells express invariant receptors for these and the second being “cognate” signals which require TCR-driven signaling upon binding to cognate peptide-major histocompatibility complex (pMHC)^13,19,20^. While these requirements vary with CD8 and CD4 T cells, previous studies, including ours suggest that spMHC are also critical even when homeostatic cytokines (the public factors) are broadly available^20–22^. The relative contributions of these two sets of signals are still being dissected. It appears that within a defined niche in which T cells compete for trophic factors, publicly available signals such as cytokines are less vital for survival and maintenance compared to access to private specific spMHC-TCR interactions^20,23,24^. Several studies have illustrated the effect by which T cells with a certain TCR can prevent transferred T cells of the same TCR specificity from undergoing homeostatic proliferation, indicating that the competitive variable is access to a specific spMHC^23,24^. Other studies have corroborated this effect by showing that blocking TCR-spMHC interactions even in the absence of foreign antigen results in poor T cell maintenance^25,26^. There are, however, studies that question the role of spMHC on T cell homeostasis and survival; some studies have demonstrated that survival without MHC is possible for more differentiated, or memory, T cells compared to naïve^27–30^. However, it is plausible that for these cases, spMHC interactions may have an alternative effect rather than simply boosting survival, and this has yet to be explored. Taken together, therapeutically manipulating bulk populations of T cells in vivo would require a combination of strategies. In this context, although we can modify the levels of cytokines in the body by exogenous administration (as discussed above), there is as yet no clear approach available to globally increase spMHC and thus tweak the population dynamics of peripheral T cells. In the case of CD4 T cells, however, bulk of the relevant spMHC are likely to be presented by hematopoietic cells, specifically by APCs. Therefore, one potential strategy to increase all spMHC would be to increase the number of APCs in the body itself.

Among the different molecules that help promote maintenance and differentiation of APCs, Fms-like tyrosine kinase 3 ligand (Flt3L) is a crucial growth factor, both within and outside of lymphoid tissue^31^. Recently, Flt3L has been explored as a therapeutic factor to (1) promote tumor antigen uptake, presentation, and availability to activate cognate T cells^32–34^, and (2) potentiate vaccine-induced immunity to foreign antigen^35,36^. Additionally, Flt3L, among other leukocyte-expanding cytokines, has recently been explored in the context of emergency, or demand-adapted hematopoiesis; upon inflammatory events and stimuli, these cytokines can act in an acute period to increase dendritic cell expansion and production to generate a rapid immune response to the perceived threat^37,38^. However, to date, the correlative effects of such an induction of APCs and APC progenitors on populations of T cells, have not fully been elaborated. Flt3L has also been previously observed as a potential enhancer of homeostatic peripheral expansion of reconstituted T cells in lymphopenic mouse models, that primarily demonstrate the effects on adoptively transferred T cells to a thymectomized host^39^. Other than one study^40^ which briefly examined the broad impact of recombinant Flt3L has on memory populations in an intact host, the subsets and markers of T cells that arise with Flt3L administration remain unclear.

In this study, we aimed to determine the effects on T cell populations of administering exogenous Flt3L to wild type C57BL/6 mice, in the absence of any additional antigen. We examined different subsets of memory and naïve T cells using phenotypic as well as functional analyses after Flt3L treatment. Our results reveal several interesting phenomena. Firstly, our model corroborated the effects of Flt3L increasing the populations of APCs and APC progenitors. Secondly, we find that there was an additional effect in both the CD4+ and CD8+ T cell compartments. Specifically, we observed a transient increase in the population of CD44-low, CD62L-low T cells, as well as an increase in the subsets of T cells expressing KLRG1 and CD25. Finally, we show that the CD44-low, CD62L-low T cell population, particularly the CD8+ T cell subset, represents a poorly understood subset of the homeostatic T cell pool that is capable of significant IFNγ and TNFα production upon ex vivo stimulation. These findings are likely to be consequential in not only understanding the impact of mouse experiments (and perhaps human studies) involving Flt3L treatment in vivo, but also in appreciating the functional significance of a transitory subset of effector-like CD8+ T cells.

## Results

### Administration of Flt3L modifies the proportions of antigen-presenting cells

**Figure 1:**
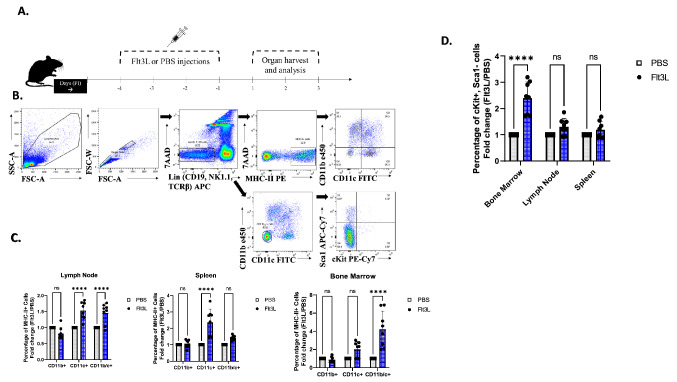
FL causes an increase in percentage of APC subsets and APC progenitors. (**A**) Experimental design as follows: 18 B61X2 mice were grouped: 9 were injected with 10 µg/100 µl of FLT3L, and the remaining 9 with 100ul of PBS for 4 consecutive days. Injections were performed intraperitoneally. After the 4th injection (labelled PI = post injection) , 8 mice/group were sacrificed on day 1 PI, and 3 mice sacrificed/group each subsequent day. Bone marrow, spleen, and lymph nodes were isolated, crushed, and stained with fluorescent markers. (**B**) Representative dot plots from the lymph node illustrating the gating strategy for CD11b and CD11c (top), and Sca1 and cKit (bottom). (**C**) Day 1 post-IP injection results of the DC subsets as a percentage of MHC-II+ cells, shown from Lymph Nodes (left), Spleen (middle), and Bone Marrow (right). Results are tabulated from two experiments (N = 3/group, N = 5/group) and normalized to the fold change of Flt3L-injected group/control group for each respective experiment. Grey rectangle = control group, Blue = Flt3L-injected group. (****) indicates statistical significance, P value ≤ 0.0001. (**D**) Day 1 post-IP injection results of cKit+, Sca1− cells from the Bone Marrow, Lymph Nodes and Spleen. Results are tabulated from two experiments (N = 3/group, N = 5/group) and normalized to the fold change of Flt3L-injected group/control group for each respective experiment. Grey rectangle = control group, Blue = Flt3L-injected group. (****) Indicates statistical significance, P value < 0.0001.

In order to arrive at a regimen of Flt3L that alters APC frequencies in an intact host, we first monitored APC and APC progenitor populations with intraperitoneal injections of Flt3L. Wild type C57BL/6 mice were injected with either PBS (1% Bovine Serum Albumin [BSA]) or Flt3L (10 μg/100 μl) for 4 consecutive days, after which groups of mice (8 mice per group) were euthanized on Days 1, 2 (3 mice per group) and 3 (3 mice per group) post-injection (PI), as illustrated in Fig. 1A. On Day 1 PI, we observed an increase in the percentage of both the CD11c+, and CD11c/b+ cells in the lymph nodes, CD11c+ cells in the spleen, and CD11b/c+ cells in the bone marrow (p < 0.0001) (Fig. 1C). Representative gating strategies are shown in Fig. 1B, and a representative flow dot-plot comparison between the Flt3L-treated group versus the PBS-treated group is shown in Suppl. Fig. S1C. These specific populational increases validate previous findings of spleen-APC expansions with the use of Flt3L, reported by several studies^40–42^. On Days 2 and 3 PI, there was no noticeable change in APC subsets in any organ, indicating that the shift observed was transient in nature (Suppl. Fig. S1A).

Flt3L not only acts as a homeostatic growth factor for APCs, but can also potentiate expansion of hematopoietic progenitor cells (HPCs)^42,43^. We measured the impact on Flt3L injection on various categories of progenitor cells, using c-Kit and Sca-1 and found an increased proportion of c-Kit+/Sca-1-cells in the bone marrow (p < 0.0001) (Fig. 1D). This is consistent with several other studies which reported changes in the bone marrow with administration of exogenous Flt3L^38,41,44,45^. Representative gating strategies for this population is shown in Fig. 1B. These progenitor cells remained increased in the Flt3L-treated bone marrow through Day 2 PI, though by Day 3 these levels were similar to the PBS treated mice (Suppl. Fig. S1B). c-Kit+, Sca-1- cells have been characterized in previous literature as belonging to the Myeloid Progenitor cell lineage^46,47^. Taken together, these results validate that the Flt3L infusion was functional in our model and led to the expected expansion of APCs in intact mice.

### Flt3L affects composition of CD4+ and CD8+ T cell subsets

**Figure 2:**
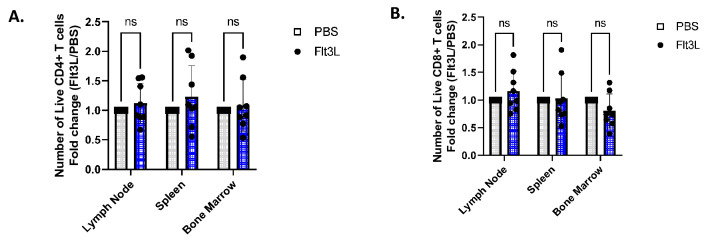
CD4+ and CD8+ T cell numbers remain stable with Flt3L injection. (**A**) Lymph node, spleen, and bone marrow samples were processed and stained to analyze CD4 numbers 1 Day PI. Grey rectangle = control group, Blue = Flt3L-injected group. (**B**) Lymph node, spleen, and bone marrow samples were processed and stained to analyze CD8 numbers 1 Day PI. Grey rectangle = control group, Blue = Flt3L-injected group.

With the changes observed in the APC compartments, we next sought to examine if there were any corresponding changes in the T cell compartments. Cells from the lymph node (LN), spleen, and bone marrow were first analyzed for the quantity of CD4+ versus CD8+ numbers. On all three days of mice examined, there was no observed change in the absolute numbers of live CD4+ or CD8+ T cells in any of the three tissue sites examined with or without Flt3L (Fig. 2; Suppl. S2B). In our flow-cytometry gating of T cells (Suppl. Fig. S2A), we first gated on TCRβ+ cells, followed by staining of CD4 and CD8, which allowed us to exclude any potential double-negative (DN) cells, or Natural Killer T cells (NKT). When comparing the fold-change of the results experiments using this gating vs. previous experiments’ gating which did not include a specific CD8 gate, our results remained largely unchanged, indicating that the DN and NKT cells are low frequency, and most likely do not contribute significantly to any of the Flt3L-induced effects.

**Figure 3:**
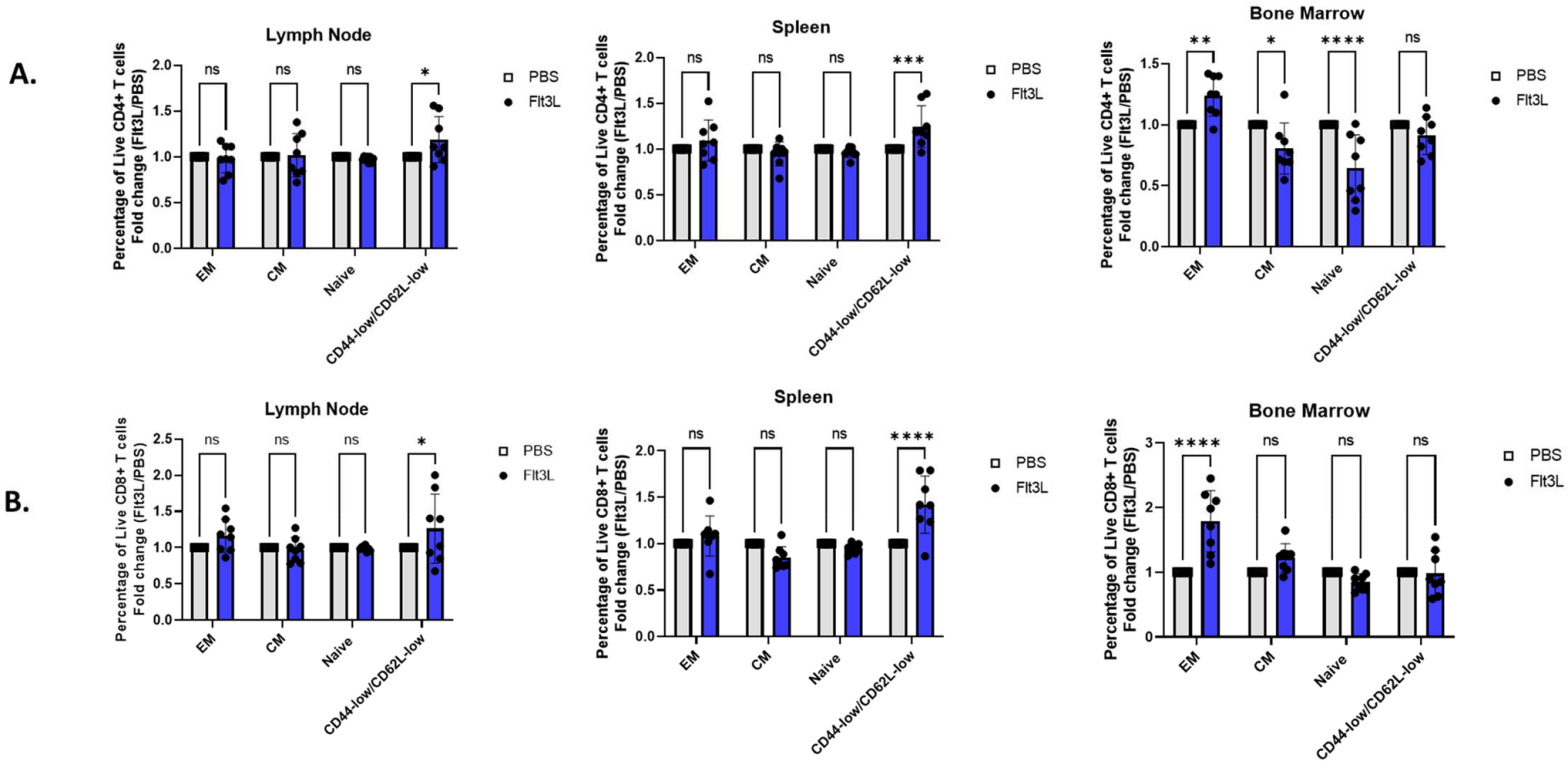
Flt3L affects composition of CD4+ and CD8+ T cell subsets. (**A**) Samples from the LN (left), spleen (middle), and bone marrow (right) were analyzed for memory subtype. CD4 central memory (CM) and effector memory (EM), naïve, and CD44-low/CD62L-low populations were assessed in proportion to respective CD4 total populations. White squares = PBS treated group, Black circles = FL (Flt3L) treated group. (*) indicates statistical significance, P value < 0.05, (**) indicates statistical significance, P value = 0.0077, (***) indicates statistical significance, P value = 0.0007, (****) indicates statistical significance, P value < 0.0001. (**B**) Samples from the LN (left), spleen (middle), and bone marrow (right) were analyzed for memory subtype. CD8 central memory (CM) and effector memory (EM), naïve, and CD44-low/CD62L-low populations were assessed in proportion to respective CD4 total populations. White squares = PBS treated group, Black circles = FL (Flt3L) treated group. (*) indicates statistical significance, P value = 0.0392, (****) indicates statistical significance, P value < 0.0001.

While the absolute number of CD4+ or CD8+ T cells did not change with Flt3L administration, it remained conceivable that subsets within these T cell compartments were altered. Using the canonical surface markers associated with memory phenotypes in mice, CD44 and CD62L, we first looked at the relative percentages of different CD44/CD62L T cell subsets: CD44-hi, CD62L-hi cells are typically classified as Central memory (T_CM_), CD44-hi, CD62L-low cells as Effector memory (T_EM_), and CD44-low, CD62L-hi cells as Naive (T_N_) (Suppl. Fig. S2A). Among the CD4+ T cells from the lymph nodes and spleen, there was no difference on Day 1 PI in the T_CM_ or T_EM_ or naïve populations in mice that received Flt3L versus PBS (Fig. 3A). Strikingly, however there was a significant increase in the proportion of CD4+ CD44-low/CD62L-low cells in these tissues on Day 1 PI (lymph node p < 0.05, spleen p = 0.0007) (Fig. 3A). In the bone marrow, there was an observed decrease in the proportion of T_CM_ and naïve CD4+ T cells, with a corresponding increase of T_EM_ cells (T_EM_ p = 0.0077, T_CM_ p < 0.05, Naïve p < 0.0001) (Fig. 3A) In the CD8+ T cell population, there appeared to be an increase in the percentage of the CD44-low/CD62L-low cells in the lymph node and spleen ( lymph node p = 0.0392, spleen p < 0.0001) (Fig. 3B). Similar to the CD4+ T cell compartment, in the bone marrow on Day 1 PI there was a significant increase in the proportion of T_EM_ cells (Fig. 3B). Overall, our results indicate a significant shift in CD4+ and CD8+ T cell subsets after Flt3L administration, with an increase in effector memory T cells in the bone marrow, and an increase in CD44-low,CD62L-low T cells in the lymph nodes and spleen.

### FLT3L acutely enhances KLRG1 and CD25 expression

**Figure 4:**
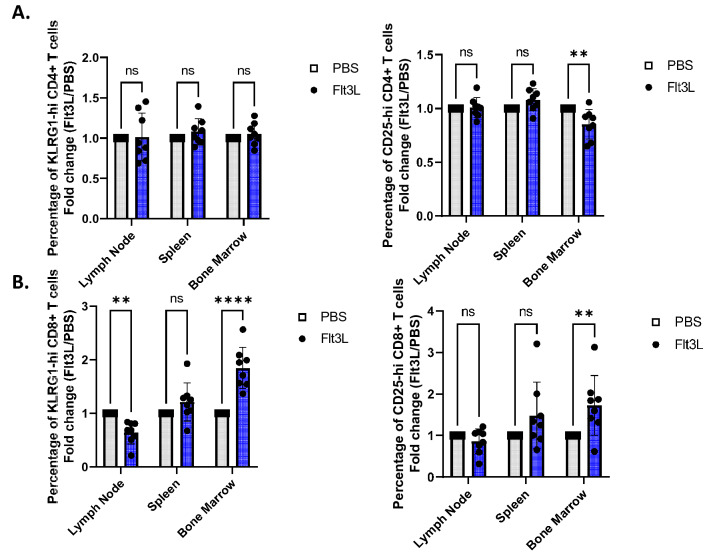
FLT3L transiently enhances KLRG1 and CD25 expression. (**A**) The percentage of KLRG1-high (right) and CD25-hi (left) cells as a proportion of total CD4+ T cells in the lymph node, spleen, and bone marrow on Day 1 PI. Grey rectangle = control group, Blue = Flt3L-injected group. (**) indicates statistical significance, P value = 0.0026. (**B**) The percentage of KLRG1-high (right) and CD25-hi (left) cells as a proportion of total CD8+ T cells in the lymph node, spleen, and bone marrow on Day 1 PI. Grey rectangle = control group, Blue = Flt3L-injected group. (**) indicates statistical significance, P value < 0.01. (****) indicates statistical significance, P value < 0.0001.

While evaluating additional panels of T cell markers, we observed two striking phenotypes within the CD4+ and CD8+ T cell compartment. First, we observed that the marker killer cell lectin-like receptor subfamily G member 1 (KLRG1), which is a marker of T cell activation and differentiation^48,49^, was increased (p < 0.0001) in the CD8+ T cells from the bone marrow of mice that received Flt3L (Fig. 4B). This increase was not observed for the CD4+ T cells in the bone marrow (Fig. 4A). Interestingly, this increased KLRG1 was transient, and was not present on either Day 2, or Day 3 PI (Suppl. Fig S3). Another marker, CD25, was also observed to follow a similar pattern as KLRG1 for CD8+ T cells, demonstrating a significant, though transient increase in the CD25-hi cells in the bone marrow (p < 0.01) (Fig. 4B; Suppl. S3). Interestingly, for CD4+ T cells, there was a significant decrease in these CD25-hi cells in the bone marrow (p < 0.01) (Fig. 4A). Overall, these differences highlight a transient phenotypic change skewing towards activation in the CD4+ and CD8+ T cell compartment with the administration of Flt3L.

### CD44-low/CD62L-low T cells can produce inflammatory cytokines

The transient increase in CD44-low, CD62L-low T cells with Flt3L exposure prompted us to examine this population on a more functional level. Although much work has been done to define other subsets of the CD44/CD62L axis, there is very little information on characterizing the CD44-low, CD62L-low subset. To explore these cells, we cultured cells from the bone marrow and spleens of wild type C57BL/6 mice on plates coated with either anti-CD3 or PBS. After 2 h of culture, we harvested the wells and prepared them for intracellular cytokine staining (ICS), which was analyzed using flow cytometry. The gating strategy for analyzing the CD44/CD62L T cell subsets is shown in Supplemental Figure S4A. Strikingly, there was a large shift with the CD4+ and CD8+ T cells treated with anti-CD3 into the CD44-low, CD62L-low and effector memory compartments in both the spleen and bone marrow (though not in the CD4+ bone marrow) (Suppl. Fig. S4B).

**Figure 5:**
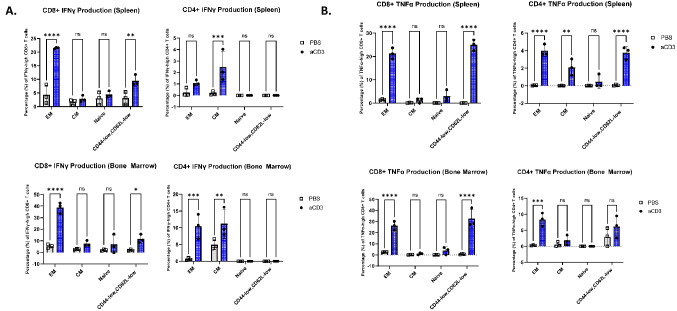
CD44-low/CD62L-low T cells can produce inflammatory cytokines. (**A**) The percentage of IFNγ-high CD8+ (left) and CD4+ (right) T cells within each compartment of CD44/62L gate in the spleen and bone marrow. Grey rectangle = PBS-treated, Blue = αCD3-treated. (*) indicates statistical significance, P value = 0.0241. (**) indicates statistical significance, P value < 0.01. (***) indicates statistical significance, P value < 0.001. (****) indicates statistical significance, P value < 0.0001. (**B**) The percentage of TNFα-high CD8 + (left) and CD4+ (right) T cells within each compartment of CD44/62L gate in the spleen and bone marrow. Grey rectangle = PBS-treated, Blue = αCD3-treated. (**) indicates statistical significance, P value = 0.0012. (***) indicates statistical significance, P value = 0.0003. (****) indicates statistical significance, P value < 0.0001.

When we examined cytokine production for these different subsets, we observed a significant increase (spleen p < 0.0001, bone marrow p < 0.0001) in interferon gamma (IFNγ) production among the effector memory population, as well as the CD44-low, CD62L-low population of CD8+ T cells (spleen T_EM_ p < 0.0001, spleen CD44-low/CD62L-low p < 0.01, bone marrow T_EM_ p < 0.0001, bone marrow CD44-low/CD62L-low p = 0.0241) that received anti-CD3 stimulation (Fig. 5A). This was in contrast to the CD4+ stimulated group, in which central and effector memory cells were the major contributors to the IFNγ production in the spleen and bone marrow (spleen T_CM_ p < 0.001, bone marrow T_EM_ p < 0.001, T_CM_ p < 0.01). Furthermore, the CD44-low, CD62L-low CD4+ and CD8+ population demonstrated comparably increased amounts of TNFα production with anti-CD3 stimulation to the effector memory population, in the spleen (p < 0.0001) and CD8+ T cells alone in the bone marrow (p < 0.0001) (Fig. 5B).

It was possible that the CD44-low/CD62L-low cells being stimulated as outlined above represented a population of cells that were transitioning into becoming CD44-high effector memory cells and downregulated CD62L, thereby confounding our results^50^. To circumvent this, we sorted unactivated cells from the spleens of wild type B6 1 × 2 mice into the four populations of CD44/CD62L (Suppl. Fig. S5). After sorting, the respective populations were then stimulated with plate-bound anti-CD3 or left unstimulated with media as a control. Interestingly, we observed that the CD44-low/CD62-low population still produced IFNγ and TNFα when stimulated. This was more apparent for the CD8+ T cells, which had sorted CD44-low/CD62L-low cells produce significant amounts of IFNγ (p < 0.0001) and TNFα (p = 0.0007) compared to the non-stimulated controls (Suppl. Fig. S5A + C). In both cases, this was significantly higher than production of cytokines from Naïve CD8 T cells, in which there was no significant IFNγ production when stimulated, and a slight increase of TNFα (p = 0.0174). For the CD4+ T cells, there was a slight increase of the CD44-low/CD62L-low cells in IFNγ and TNFα production when stimulated, though both cytokine productions were not statistically significant (Suppl. Fig. S6B + D). Ultimately these results show that for the CD8+ T cells, the CD44-low/CD62L-low cells are a functionally significant group that can contribute to an inflammatory response.

## Discussion

In this paper, we have analyzed changes to the T cell compartment of an intact host with the exogenous administration of Flt3L. This is important, since most studies to date use lymphopenic or irradiated hosts to evaluate the consequences of Flt3L as an expander of APC and T cell populations^39,40^. In intact hosts, Parajuli et al. reported in 2001, that the use of Flt3L in intact mice resulted in an expansion of splenic CD11c+, and CD11b/c+ cells, which we recapitulate in our findings^40^. In addition to our observations with APCs, we also find the expansion of HPCs in the bone marrow consistent with the idea that Flt3L plays an important role in acting as a differentiator and growth factor along the DC developmental pathway^51,52^. Recent studies such as Lin et al. have also highlighted this importance of exogenous Flt3L administration on the downstream lineage expansion of APCs and early APC progenitor populations^38^. Our study not only corroborates certain important findings from that paper (namely the expansion of the myeloid progenitor population, particularly in the bone marrow), but attempts to explore these effects beyond the myeloid compartment.

One key focus was to examine any relational changes within the T cell compartment, particularly to memory T cells. The motivation for this work was hypothesis-driven. Based on our studies on the role of self-peptides and related signaling on T cell homeostasis, we suggested that altering the abundance of overall antigen-presentation in vivo would help augment memory T cell turnover or maintenance^18,53–55^. While is it not possible to alter all self-peptides one at a time, we explored here the impact of a global regimen using FLT3L. If successful, this would be a viable way to perhaps change the turnover of memory T cells even in special circumstances such as latent HIV infection^54^. Interestingly we observe a small flux of memory phenotype CD8 T cells within a narrow subset but not an overall perturbation of the steady state. In addition to memory T cells, it is also important to consider shifts in the naïve T cell population, as these cells also derive some basal homeostatic signaling from interaction with spMHC^56,57^. Curiously, we observed a decrease in the proportion of naïve T cells with Flt3L in the CD4+ from the bone marrow. Because the number of T cells tabulated did not change with or without Flt3L, it is plausible that a compartmental shift in the bone marrow occurred, with a higher proportion becoming effector memory from the actual naïve compartment. Furthermore, actual differences in the clonality of naïve T cells should be considered, as several studies have addressed the relation between spMHC interaction and changes in the T cell repertoire^20,58^.

A significant change was in the CD44-low/CD62L-low population of cells, and a decrease in the bone marrow CD4+ central memory cells. The CD44-low/CD62L-−population is a subset that has not received a lot of attention; though some reports have referred to them as “double negative”, their role is not clear^59,60^. The functional characteristics of the CD44-low, CD62L− T cell population, evaluated by cytokine production after ex vivo stimulation, revealed some intriguing features. First, we observed that even a brief in vitro stimulation resulted in an increase in the percentage of CD44-low, CD62L− CD8+ T cells in both the spleen and the bone marrow, but for CD4+ T cells, we only observed a significant increase in the spleen, though in the bone marrow there was a visible increase in the proportion of these cells with Flt3L. Secondly, we found that these cells in the CD8+ population, upon anti-CD3 stimulation, they were able to produce significant amounts of IFNγ and TNFα, comparable to the production of effector memory cells, and much greater than naïve or central memory cells. This was in contrast to the major cell types producing these cytokine in the CD4+ T cell compartment with stimulation; while the splenic and bone marrow CD4+ CD44-low/CD62L-low cells produced increased amounts of TNFα, the effector and central memory cells were the major IFNγ producers. IFNγ and TNFα are two important effector cytokines secreted by T cells typically requiring differentiation and T cell help^61,62^. Particularly, it has been observed that CD8+ T cells with high CD44 expression generate high amounts of these cytokines, and corresponds with a robust inflammatory effector response^61,63^. It was therefore curious that this population of CD44-low, CD62L-low CD8+ T cells would also produce these cytokines in response to anti-CD3 stimulation. It was possible that we are observing a subset of CD8+ T cells that are transitioning into becoming effector memory cells, but have not upregulated CD44; nevertheless, in our experiments in which the cells were pre-sorted before plate-bound activation, there was still increased cytokine production of the CD8+ CD44-low/CD62L-low population. In studies characterizing human CD8+ T cell populations, there is a subset defined as T_EMRA_, which is identified as being CD45-RA+, CCR7−, qualities which distinguish it from other memory and naïve T cells, and yet this population also produces Th1 cytokines^6,64^. Perhaps the CD44-low CD62L-low population represents a T_EMRA_-like population of CD8+ T cells in mice.

Furthermore, while writing this current manuscript, a study by Nakajima et al. was published elaborating the relevance of CD44-low, CD62L− CD8+ T cells^65^. In their work, they describe this population as being important cells with effector-like properties, particularly in the rejection of tumors. We have shown in our studies data consistent with this finding, namely that more IFNγ was produced by CD44-low CD8+ T cells, than even the CD44-hi population. Nakajima et al. also found that this double-low population of cells seemed to arise from the naïve subset (CD44-low, CD62L+), and eventually transition into effector memory cells (CD44/CD62L-hi). This appears to synergize with our data shown in S4B, where, at least for the spleen, there is a seeming shift from the naïve compartment into the double-negative compartment. Finally, Nakajima et al. found that the induction of this double negative population was impaired in aged mice, but could be recovered by increased exposure to nonself antigen. Our present study complements these findings in several ways: (1) we have identified that these double negative cells are capable of producing effector cytokines, and (2) the use of Flt3L should increase major antigen presenting cells, which would theoretically boost access to both self and nonself pMHC complexes. Although our model did not involve exposure of antigen in addition to exogenous Flt3L, it is possible that existing self-pMHC complexes have an important role in at least transiently boosting this double negative population.

We also observed a transient increase in the surface expression of KLRG1 and CD25 on CD8+ T cells in the bone marrow of mice that received Flt3L. KLRG1 and CD25 are both activation markers involved in homing and responsiveness to IL-2 respectively. The specific relationship to FLT3L here needs further study. While we equate this to a consequence of transient increase in endogenous antigen presentation, tying these to APCs and MHC will require future studies ablating these. In an intact animal such perturbations are hard to achieve (since APC ablation by DT-based systems of MHC-deletion by Cre-Lox approaches, all have impacts on the overall milieu). We also highlight the acute nature of our findings. The administration of Flt3L to the mice was done for 4 days; while other studies have extended this duration of injection, we found that our minimum duration of Flt3L injection could still elicit an increase in APC and APC progenitor populations. Acute effects on T cell populations warrant attention; a recent study by Moreews et al. found a transient increase in a population of activated T cells associated with cases of the SARS-CoV2-associated Multisystem Inflammatory Syndrome in Children (MISC)^66^. In our study, it is unclear if continuing the Flt3L treatment would indeed result in more extended effect on T cells. Previous work from Brasel et al. at the same dose of Flt3L we used (10 μg), the authors found that after 3 days of treatment, the number of progenitors in the bone marrow reached a maximal expansion amount^67^. Further work into evaluating the in vivo half-life and kinetic impact of exogenous Flt3L should be performed.

A limitation of our study is that we cannot explicitly infer whether the changes we have seen in the T cell compartment are indirectly through APC expansion, or through direct action by Flt3L on T cells. However, to date there is no evidence that T cells themselves express the Flt3L receptor Flt3, although further confirmatory experiments involving staining of this protein are warranted.

In sum, we report in this study a new examination of T cell compartmental shifts due to corresponding changes in APCs. We find that although the size of CD4+ and CD8+ T cells themselves did not change numerically, there were several functional phenotypic shifts both in frequency of memory subsets, and in expression of surface receptors. We have also demonstrated that an observed increase in CD44-low, CD62L-low T cells may be important in the context of inflammatory cytokine production. In addition to providing data on the healthy-state homeostasis of T cells, we hope to add to the growing literature on the use of Flt3L and similar APC-influencing tools in cancer therapy and vaccine development.

## Materials and methods

### Mice and Flt3L/PBS injections

C57BL/6J and B6.SJL-Ptprc^a^Pepc mice from Jackson Laboratories, and B6NTac and B6.SJL-Ptprc^a^/BoyAiTac from Taconic Biosciences were obtained and bred in a sterile housing facility. These mice were bred for dual congenic expression of CD45.1/45.2 using males and females from opposing origins of purchase to avoid strain drift. All resulting B6 CD45.1 × 2 mice used for this study were both male and female, between the ages of 6–16 weeks old. For the intraperitoneal injections (n = 28), 100μL of either 10 μg of Flt3L dissolved in sterile 1 × PBS with 1% Bovine Serum Albumin (BSA), or 100μL sterile 1 × PBS with 1% BSA (as the control group) was injected, and mice were randomly selected to be in either the Flt3L (n = 14) or control group (n = 14). B6 CD45.1 × 2 mice (n = 3) used for the anti-CD3 in vitro assay did not receive any injections, and were euthanized, and samples processed according to Step 2 detailed below. This study was carried out following all the relevant guidelines and regulations. Housing of mice, handling, intraperitoneal injections, euthanasia, and tissue isolation, were all done in accordance to our institutionally approved IACUC protocol (UMB #1119010). Author GW performed the injections, and GW and AG contributed equally to the euthanasia and tissue dissection. ARRIVE guidelines for description and presentation of live animal studies were consulted and incorporated into this manuscript.

### Cell isolation and preparation

Single cell suspensions were prepared from isolated lymph nodes, spleens, and bone marrow of all sacrificed mice. For the lymph nodes and spleens, they were separately dissected and filtered through 40 μM nylon mesh with cold Crushing Buffer (1 × PBS, FCS, and antibiotic–antimycotic solution). Bone marrow was isolated from the femur, tibia, and fibula of mice by removal of the bones, incision at separate ends of the bones, and flushing of cold Crushing Buffer through the medullary cavity using a 26-gauge syringe. Cells from each tissue were spun at 1200 RPM for 7 min, resuspended in cold Crushing Buffer, and counted using a hemocytometer. GW and ZF contributed equally to the cell isolation and counting of cells isolated from tissues.

For the isolation of tissues from mice that received Flt3L injections, aliquots of 5 million cells were separated to be used in the various flow panels as described in Step 3. For the anti-CD3 stimulation assay, cells were further purified; the single cell suspensions were added to 15 mL polystyrene round bottom tubes with Ficoll and spun at 2000 RPM for 10 min at room temperature. The buffy coat portion from each tube was collected, washed with Crushing Buffer, and spun at 1200 RPM for 7 min at 4°. The cells were resuspended in T Cell Medium (RPMI, Glutamine, Antibiotic–antimycotic, FCS, and 2-mercaptoehtanol) and counted for the anti-CD3 stimulation assay as described in Step 4.

### Flow cytometry for in vivo Flt3L/PBS injections

Cells prepared by Step 2 were spun and resuspended in Fc-block (a cocktail of mouse, rat, and hamster IgG, as well as anti-CD16/32 Rat anti-mouse IgG clone 2.4G2) for 15 min in 4°. After this incubation, we stained each sample with a cocktail of antibodies, and incubated the samples in the dark for 15 min in 4°. The panels described in this paper for the analysis of mice injected with Flt3L or PBS incorporated the following antibodies (target + clone): For CD4 and CD8 quantification and memory differentiation, we used CD4 (RM4.5 and GK1.5), CD8β (H35-17.2), CD44 (IM7), CD62L (MEL-14), TCR-beta (H57-597). For DCs and DC progenitors, we used a lineage stain (CD19 [1D3], TCR-beta [H57-597], and NK1.1 [PK136]), MHC-II (M5/114.15.2), CD11b (M1/70), Sca1 (D7), CD11c (N418), cKit (2B8). Information on fluorophore, manufacturer, and dilution of antibody can be found in Supplemental Table 1. Viability staining was performed using 7-Actinomycin D (7AAD). For staining of CD4 and CD8, we used CD4 (RM4.5 and GK1.5), CD8β (H35-17.2), TCR-beta (H57-597), KLRG1 (2F1), CD25 (PC-61). Following incubation with the fluorescent antibodies, they were washed and resuspended in 1 × FACS buffer (BSA, EDTA, Sodium Azide, and 1 × PBS). Samples were acquired on a BD-LSR II Flow cytometer, and analysis performed using FlowJo software, version 10. While some studies use collagenase treatment to isolate monocytes and APCs from other tissue compartments, we find that this technique does increase yield by 2–3 but does not change the relative frequencies. Avoiding collagenase also increased the reliability of staining with various flow-markers.

### Anti-CD3 in vitro assay for unsorted cells

24 h before use, a 96-well flat bottom plate was incubated with wells containing either 10ug/ml of anti-CD3ε (clone 145-2C11, Biolegend), or 1 × PBS at 37 degrees. After 24 h, the plate was washed with 1X PBS, and then incubated with T Cell Medium (RPMI, Glutamine, antibiotic–antimycotic solution, FCS, and 2-mercaptoethanol) for 10 min at 37°. After this, the T Cell Medium was removed, and 1 million T cells from the spleens and bone marrow of wild type mice prepared by Step 2 were added to each well and incubated for 2 h at 37°. After the stimulus, 10 ng of pre-warmed Brefeldin A was added to each well, and the plate was incubated for an additional 4 h at 37°. After this, the cells were prepared for intracellular cytokine staining as described in Step 5.

### Intracellular cytokine staining for unsorted cells

Cells from Step 4 were transferred to 5 ml round bottom polystyrene FACS tubes and incubated with Fc-block for 15 min at 4 °C. After this incubation, the surface antibody stains were applied. The surface panels described in this paper incorporated the following antibodies (target + clone): CD4 (GK1.5), CD8a (53-6.7), CD44 (IM7), and CD62L (MEL-14). Information on fluorophore, manufacturer, and dilution of antibody can be found in Supplemental Table 2. Viability staining was performed by using Zombie-NIR. After the surface staining for 15 min at 4°, the tubes were spun and washed with 1X FACS buffer and incubated with BD Cytofix/Cytoperm solution for 20 min at 4° in the dark. After this, the cells were spun and washed again with 1X FACS buffer and incubated with 1 × eBioscience Fixation/Permeabilization solution overnight at 4 degrees. The following day, the tubes were spun and incubated with 1 × eBio Perm Fc- Block solution for 15 min. After this, cells were stained for intracellular cytokine production: for intracellular cytokine staining, we used IFNγ (XMG1.2), and TNFα (MP6-XT22). Information on fluorophore, manufacturer, and dilution of antibody can be found in Supplemental Table 2. Cells were incubated with fluorophores for 1 h at 4° in the dark. After this period, they were washed twice with 1 × eBio Perm Buffer, and eventually resuspended in 1 × FACS buffer for analysis. Flow Samples were acquired using the Cytek Aurora and analysis performed using FlowJo software, version 10.

### Cell sorting

B61 × 2 spleens were prepared as described in Step 2. Cells were purified using Ficoll-HiPaque Density Gradient to isolate lymphocytes. Cells were washed with Crushing Buffer for 7 min at 1200RPM, after which they were incubated for 15 min with Fc-block (made with crushing buffer rather than FACS buffer). Next, the cells were stained with TCR-beta (H57-597), CD44 (IM7), CD62L (MEL-14), and a non-T cell dump channel: B220 (RA3-6B2), CD11b (M1/70), MHC-II (M5/114.15.2), and NK1.1 (PK136) in the dark at 4° for 15 min Antibodies used can be found in Supplemental Table 3. Cells were then washed with crushing buffer and stained with 7AAD. Cells were sorted using the BD FACSAria II Cytometer (gating strategy shown in Supplementary Fig. S5).

### Anti-CD3 in vitro stimulation for sorted cells

10,000 cells from each fraction were stimulated using platebound anti-CD3e (clone 500A2 BD biosceiences), 100 μL of 10 ug/mL in PBS per well) and soluble anti-CD28 (2 μg/mL, clone 37.51) in T cell media. Unstimulated control wells were cultured in T cell media alone. Technical replicates were also prepared. After 2 h of stimulation, 1 × brefeldin A (5 ng) was added to each well and incubated for a further 4 h. Final well volume was 200 μL/well.

### Intracellular staining for sorted cells

Cells from Step 7 were washed and incubated with Fc block for 10 min at 4 °C before surface antibody and fixable live/dead staining for 15 min at 4 °C. Antibodies used were CD4 (GK1.5), TCR-beta (H57-597), CD8β (H35-17.2), CD44 (IM7), CD62L (MEL-14), and Zombie NIR. Cells were washed twice with FACS buffer before incubation with fixation solution (BD cytofix/cytoperm) for 20 min at 4 °C. Cells were again washed twice before overnight incubation at 4 °C with permeabilization solution (eBioscience Fixation/Permeabilization). Cells were washed with permeabilization buffer before 15 min incubation at 4 °C with Fc-block. Cytokine antibodies were added and incubated at 4 °C for 1 h. Antibodies used were TNFα (MP6-XT22) and IFNγ (XMG1.2). Information on these fluorophores is found on Supplemental Table 3. Cells were again washed twice with permeabilization buffer before resuspension in FACS buffer and analysis using Cytek Aurora spectral flow cytometer and analysis performed using FlowJo software, version 10.

## Statistics

All analyses performed were done on GraphPad Prism version 9.1.0, using a 2 way ANOVA. When relevant, we used multiple comparisons, with a Sidak Test which corrects for multiple comparisons. The level of significance for each test was set at p < 0.05.

## Supplementary Information

Supplementary Information 1.

Supplementary Information 2.

Supplementary Information 3.

## Data Availability

The datasets used and/or analyzed during the current study are all included in the manuscript. Raw data is available from the corresponding author on reasonable request.
