# Supplementary material for: Acute effects of FLT3L treatment on T cells in intact mice
Source: Sci Rep. 2022 Nov 14;12:19487. doi: 10.1038/s41598-022-24126-4 (PMC9662129; doi:10.1038/s41598-022-24126-4)

Figure S1: DC populations and cKit+, Sca1- population on Days 2 and 3 post-IP injection

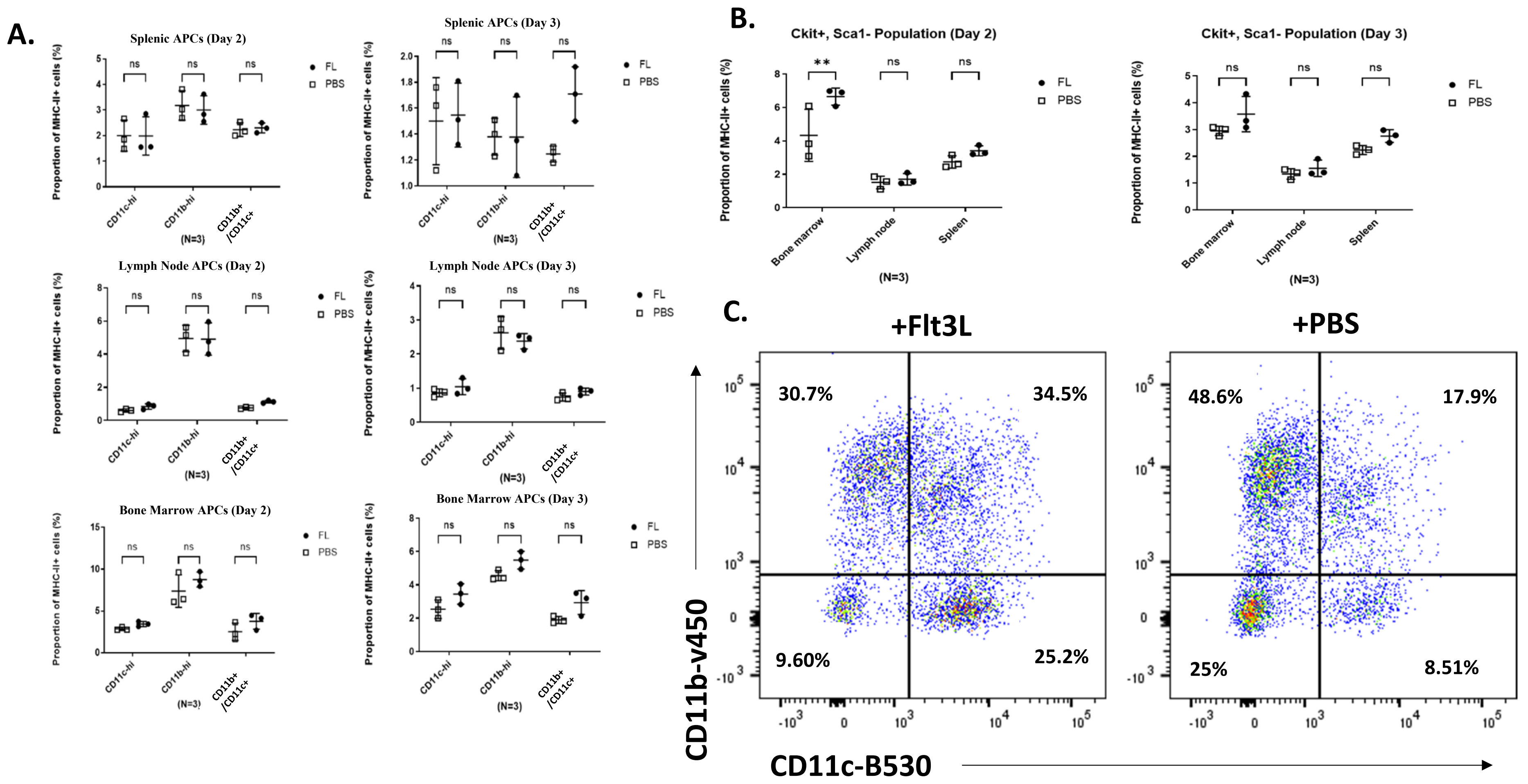

Figure S2A: Gating strategy for CD44/CD62L T cell compartments.

A.

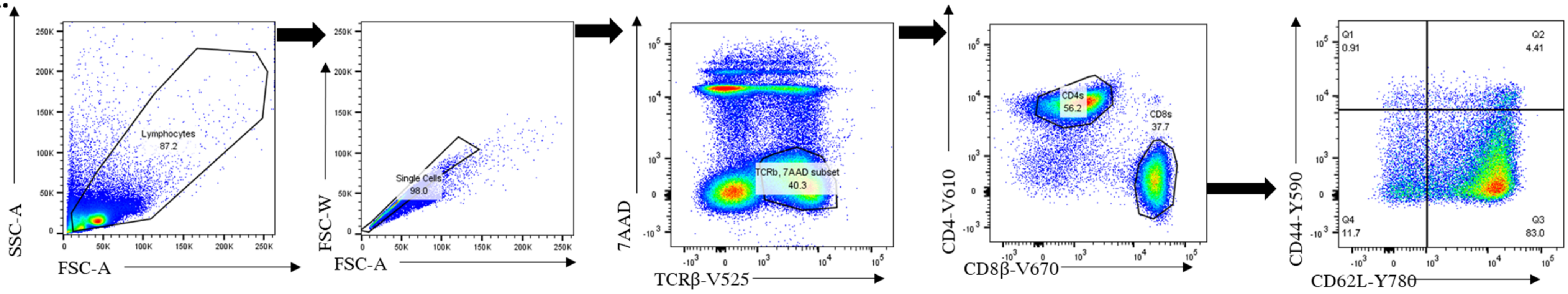

B.

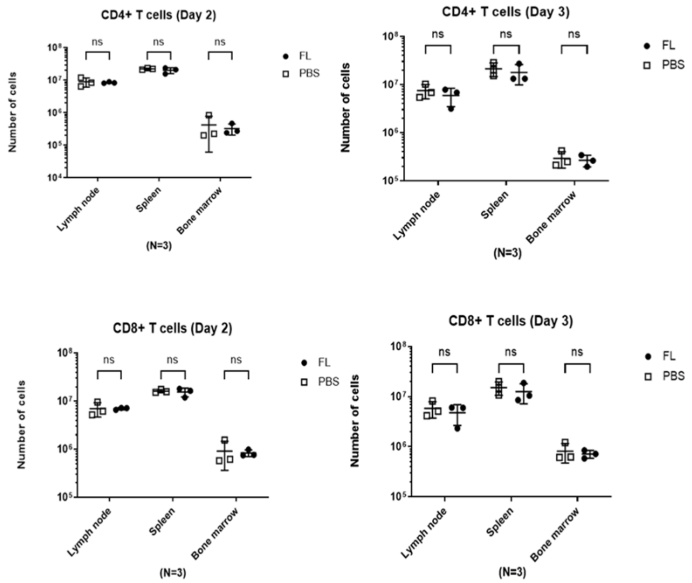

Figure S3: KLRG1 and CD25 expression on CD8 T cells

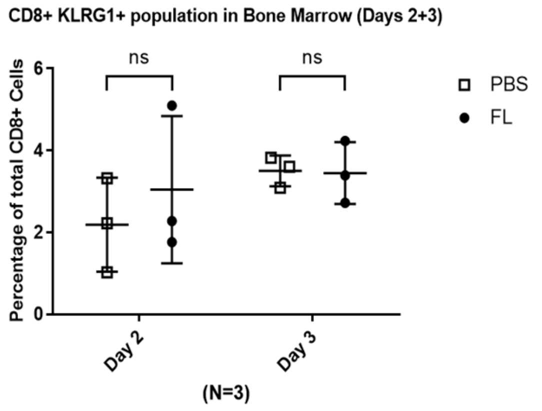

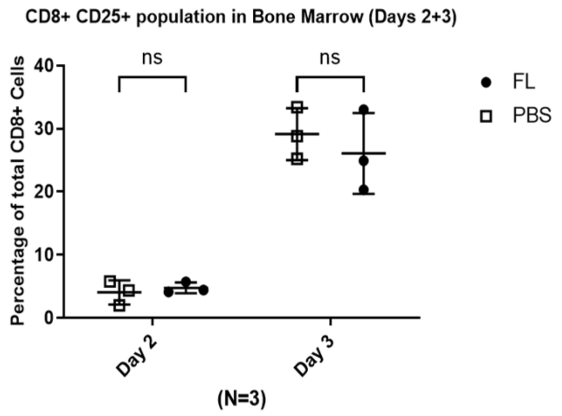

Figure S4: Gating strategy and compartmental differences for CD44/CD62L CD8+ T in vitro anti-CD3 assay

A.

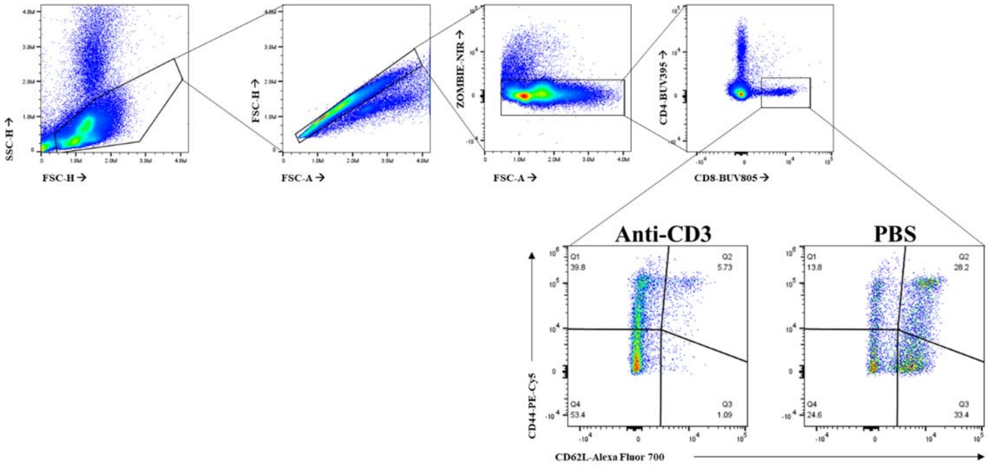

B.

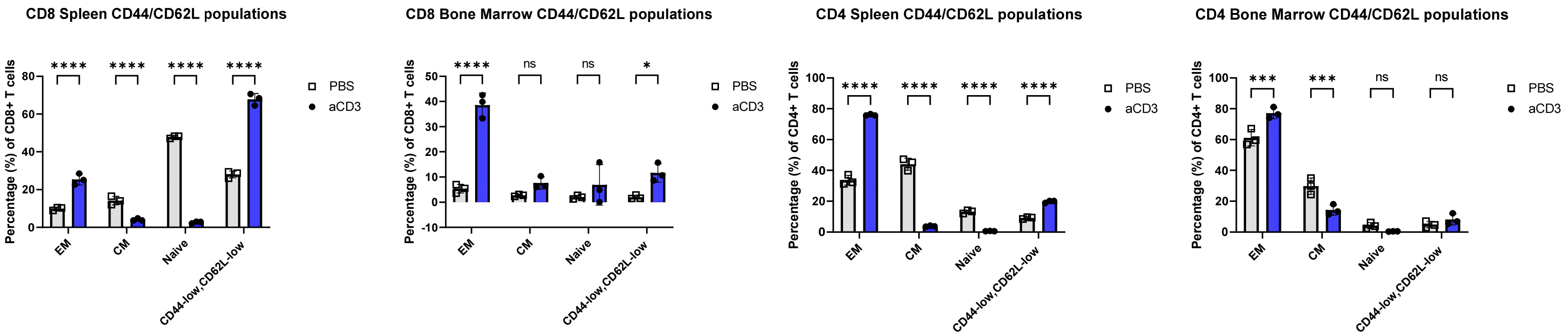

Figure S5: Gating Strategy for the Cell Sort

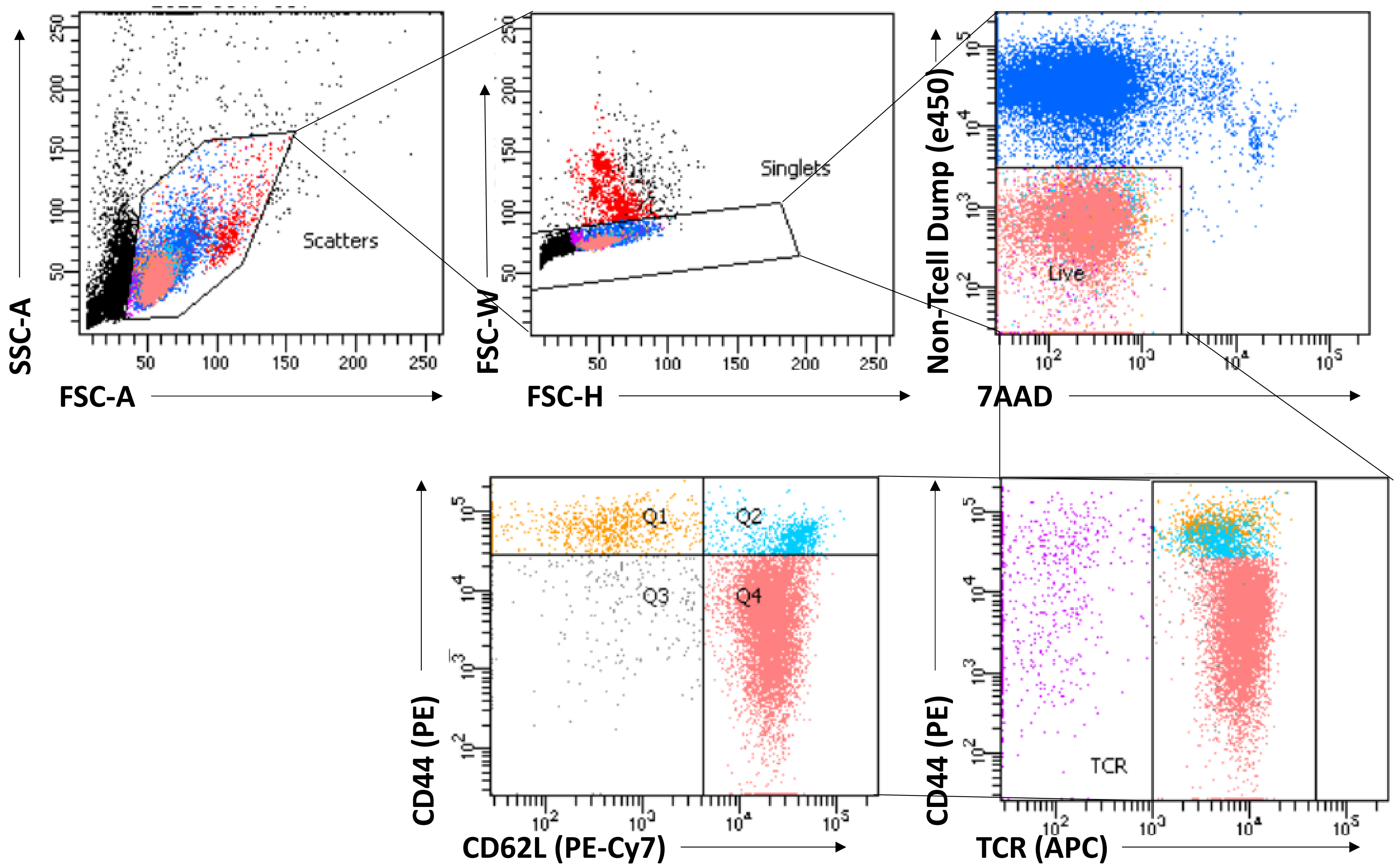

Figure S6: Sorted CD44-low, CD62L-low cells are capable of producing IFNγ and TNFα

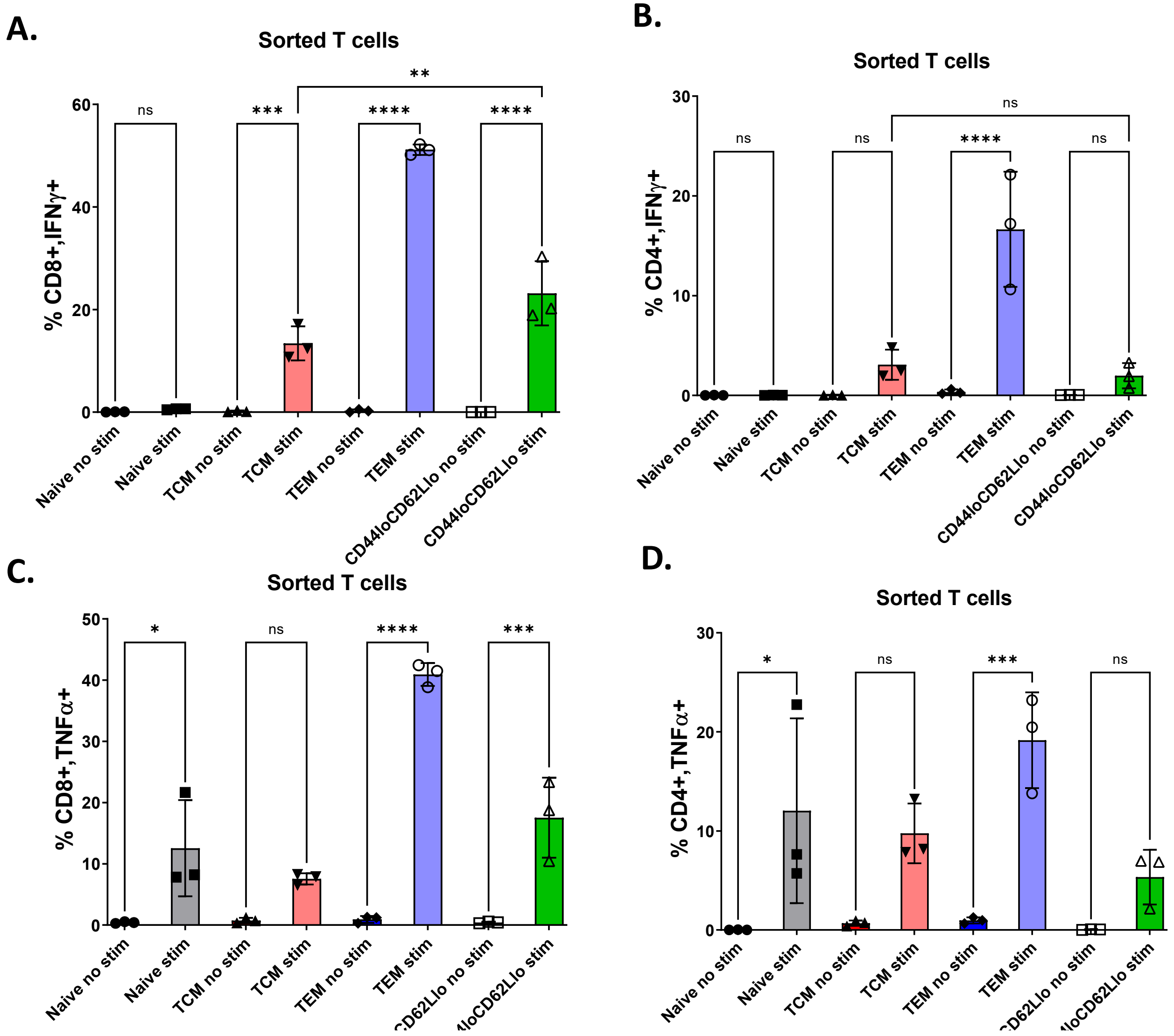

Supplement: Supplementary file 1 — Supplementary Information 1. [file 41598_2022_24126_MOESM1_ESM.pdf]
