# Supplementary material for: Acute effects of FLT3L treatment on T cells in intact mice
Source: Sci Rep. 2022 Nov 14;12:19487. doi: 10.1038/s41598-022-24126-4 (PMC9662129; doi:10.1038/s41598-022-24126-4)
Supplement: Supplementary file 2 — Supplementary Information 2. [file 41598_2022_24126_MOESM2_ESM.docx]

**Supplemental Table 1**

| **Antibody** | **Flurochrome** | **Manufacturer** | **Clone** | **Titration** |
| --- | --- | --- | --- | --- |
| CD4 | e450 | eBiosciences | RM4-5 | 1 to 500 |
| CD4 | BV605 | Biolegend | GK1.5 | 1 to 500 |
| CD8-beta | BV650 | BD | H35-17.2 | 1 to 500 |
| CD44 | PE | BD | IM7 | 1 to 300 |
| CD62L | PE-cy7 | eBiosciences/Biolegend | MEL-14 | 1 to 300 |
| TCRb | FITC | BD | H57-597 | 1 to 150 |
| TCRb | BV510 | Biolegend | H57-597 | 1 to 200 |
| TCRb | APC | Biolegend | H57-597 | 1 to 200 |
| CD19 | APC | Biolegend | 1D3 | 1 to 200 |
| NK1.1 | APC | Biolegend | PK136 | 1 to 200 |
| MHC-II | PE | eBiosciences | M5/114.15.2 | 1 to 200 |
| CD11b | e450 | eBiosciences | M1/70 | 1 to 100 |
| CD11c | FITC | eBiosciences | N418 | 1 to 100 |
| cKit | PE-cy7 | Biolegend | 2B8 | 1 to 100 |
| Sca1 | APC-cy7 | Biolegend | D7 | 1 to 100 |
| CD25 | APC-cy7 | BD | PC-61 | 1 to 200 |
| CD25 | AF700 | Biolegend | PC-61 | 1 to 100 |
| KLRG1 | PE | eBiosciences | 2F1 | 1 to 100 |
| KLRG1 | APC | BD | 2F1 | 1 to 100 |

**Supplemental Table 2**

| **Antibody** | **Flurochrome** | **Manufacturer** | **Clone** | **Titration** |
| --- | --- | --- | --- | --- |
| CD4 | BUV395 | BD | GK1.5 | 1 to 300 |
| CD8 | BUV805 | BD | 53-6.7 | 1 to 300 |
| CD44 | PE-Cy5 | eBiosciences | IM7 | 1 to 200 |
| CD62L | AF700 | Biolegend | MEL-14 | 1 to 100 |
| Zombie-NIR | Zombie NIR | Biolegend |  | 1 to 1000 |
| IFNγ | PE-Cy7 | Biolegend | XMG1.2 | 1 to 200 |
| TNFα | BV605 | Biolegend | MP6-XT22 | 1 to 250 |

**Supplemental Table 3**

| **Antibody** | **Flurochrome** | **Manufacturer** | **Clone** | **Titration** |
| --- | --- | --- | --- | --- |
| CD44 | PE | BD | IM7 | 1 to 100 |
| CD62L | PE-Cy7 | Biolegend | MEL-14 | 1 to 100 |
| 7AAD |  |  |  | 1 to 10 |
| TCRb | APC | Biolegend | H57-597 | 1 to 200 |
| CD11b | e450 | eBiosciences | M1/70 | 1 to 100 |
| B220 | e450 | eBiosciences | RA3-6B2 | 1 to 100 |
| MHC-II | e450 | eBiosciences | M5/114.15.2 | 1 to 100 |
| NK1.1 | e450 | eBiosciences | PK136 | 1 to 100 |
| Zombie NIR | NIR | Biolegend |  | 1 to 1000 |
| CD4 | e450 | BD | GK1.5 | 1 to 500 |
| CD8β | BV650 | BD | H35-17.2 | 1 to 500 |
| TNFα | BV605 | Biolegend | MP6-XT22 | 1 to 200 |
| IFNγ | FITC | Biolegend | XMG1.2 | 1 to 200 |
