# Supplementary material for: Acute effects of FLT3L treatment on T cells in intact mice
Source: Sci Rep. 2022 Nov 14;12:19487. doi: 10.1038/s41598-022-24126-4 (PMC9662129; doi:10.1038/s41598-022-24126-4)
Supplement: Supplementary file 3 — Supplementary Information 3. [file 41598_2022_24126_MOESM3_ESM.pdf]

Figure S1:

A. Proportion of CD11b, CD11c, and CD11b/c+ (DP) cells as a percentage of MHC-II+ cells in the bone marrow, lymph nodes, and spleen were evaluated on days 2 and 3 post-IP injection. White squares = PBS treated group, Black circles = FL (Flt3L) treated group.
B. Proportion of cKit+, Sca1- cells as a percentage of MHC-II+ cells in the bone marrow, lymph nodes, and spleen were evaluated on days 2 and 3 post-IP injection. White squares = PBS treated group, Black circles = FL (Flt3L) treated group. (*) indicates statistical significance, P value < 0.05.
C. Representative flow plots illustrating the difference in the CD11b/c compartments with Flt3L treatment vs. with PBS. Samples taken from the lymph node group.

Figure S2:

A. Schematic on the flow cytometry gating strategy to evaluate CD44/CD62L T cell compartments. This is showing the representative gating for CD8 memory components. CD62L+/CD44+ cells were termed "Central Memory" cells, CD62L-/CD44+ cells as "Effector memory", CD62L+/CD44- as "naïve," and CD62L-/CD44-LOW were called by their markers.
B. Lymph node, spleen, and bone marrow (right) samples were processed and stained to analyze CD4+ (top) and CD8+ (bottom) numbers on days 2-3 post-injection (Day 2 = Left, Day 3 = Right). White squares = PBS treated group, Black circles = FL (Flt3L) treated group.

Figure S3:

CD8+ KLRG1+-hi population (left) and CD8+ CD25-hi population (right) in the bone marrow at

Days 2+3 post-injection. White squares = PBS treated group, Black circles = FL (Flt3L) treated group.

Figure S4:

A. Representative dot plots illustrating the gating strategy for CD44 and CD62L, illustrating both the anti-CD3 and PBS-control conditions.
B. CD44/62L compartmental differences between anti-CD3 (white square) and PBS treated (black circle) samples in the spleen (left) and bone marrow (right). The Y axis represents the percentage of CD4+ and CD8+ T cells that are in each respective subset, as defined by CD44-LOW, CD62L- cells, effector memory (CD44+, CD62L-), central memory (CD44+, CD62L+), and naïve (CD44-LOW, CD62L+) cells. (*) indicates statistical significance, P value < 0.05. (***) indicates statistical significance, P value < 0.001. (****) indicates statistical significance, P value < 0.0001.

Figure S5:

Shown is the representative gating strategy for sorting T cells into 4 quadrants based on CD44 and CD62L expression.

Figure S6:

T cells from the spleens of WT B6 1x2 mice were sorted into 4 quadrants based on CD44/CD62L expression, as shown in Fig. S5. After sorting, each population was respectively stimulated with plate-bound anti-CD3 (stim) or left unstimulated by only adding T cell media (no stim). Cells were then harvested and stained for expression of IFNγ and TNFα. (N=3)

A. Shown are the percentages of CD8+, IFNγ-hi cells. ** represents a p value = 0.0045, ***

p value = 0.0002, **** p value <0.0001.

B. Shown are the percentages of CD4+, IFNγ-hi cells. **** p value <0.0001.

C. Shown are the percentages of CD8+, TNFα-hi cells. * p value = 0.0174, *** p value = 0.0007, **** p value <0.0001.

D. Shown are the percentages of CD4+, TNFα-hi cells. * p value = 0.0323, *** p value = 0.0008.

Supplemental Table 1: The flow cytometry panel including information on fluorophore, manufacturer, and dilution of antibody for the panel examining non-T cell and T cell populations for the Flt3L/PBS injections.

Supplemental Table 2: The flow cytometry panel including information on fluorophore, manufacturer, and dilution of antibody for the panel examining CD4+ and CD8+ T cell populations and cytokine production in the unsorted cells.

Supplemental Table 3: The flow cytometry panel including information on fluorophore, manufacturer, and dilution of antibody for the panel examining CD4+ and CD8+ T cell populations and cytokine production in the sorted cells.
